# Evaluation of Aggregate Oral Fluids for African Swine Fever Real–Time PCR Diagnostics Using Samples Collected on Romanian Farms with an Active Outbreak

**DOI:** 10.1155/2024/9142883

**Published:** 2024-08-13

**Authors:** Chungwon Joseph Chung, Marta D. Remmenga, Sarah R. Mielke, Matthew Branan, Andrei Daniel Mihalca, Oana-Maria Balmos, David Adrian Balaban Oglan, Alexandru Supeanu, Attila Farkas

**Affiliations:** ^1^ Foreign Animal Disease Diagnostic Laboratory (FADDL) United States Department of Agriculture (USDA) Animal and Plant Health Inspection Service (APHIS) Veterinary Services (VS) National Veterinary Services Laboratories (NVSL), P.O. Box 848, Greenport 11944, NY, USA; ^2^ USDA APHIS VS Center for Epidemiology and Animal Health (CEAH), Fort Collins 80526-8117, CO, USA; ^3^ University of Agricultural Sciences and Veterinary Medicine Cluj-Napoca, Cluj-Napoca, Romania; ^4^ Fatrom Aditivi Furajeri S.R.L., Pantelimon 077145, Romania; ^5^ National Sanitary Veterinary and Food Safety Authority (ANSVSA), București, Romania; ^6^ University of Agronomic Sciences and Veterinary Medicine of Bucharest, Bucharest, Romania; ^7^ Carthage Veterinary Service, P.O. Box 220, Carthage 62321, IL, Tunisia

## Abstract

African swine fever (ASF), caused by African swine fever virus (ASFv), is a highly contagious disease of domestic and wild pigs with a mortality rate that can reach 100%. Continuous spread of the virus into ASF-free regions, including the Caribbean island of Hispaniola in 2021, is causing socioeconomic burdens and presents a threat to food security. Pork-producing countries, including the United States and Canada, are urgently looking for efficient tools for early detection to reduce spread of the virus in the event of an outbreak. Previous experimental infection studies evaluated the utility of aggregate porcine oral fluids (OFs) as a sample type with a highly sensitive ASFv real-time PCR for individual blood and tissue-based diagnosis. In this study, real-time PCR with porcine OFs was further evaluated to better understand diagnostic performance using samples from three Romanian farms with an ongoing ASF outbreak. In this limited dataset using a Bayesian latent class model, no statistical difference in diagnostic sensitivity was found between the real-time PCR using aggregate OFs and the process of determining pen disease status by testing individual blood samples collected from a subset of pigs from the same pen. Known negative aggregate OF samples from pigs in the United States had no occurrences of false positives, suggesting reliable diagnostic specificity of the sample matrix used for this study. Until results are produced from further studies with sufficient sample size, aggregate OF testing using real-time PCR could cautiously be used as a supplementary sample type for ASF diagnosis alongside currently approved sample types, including blood and lymphoid tissues.

## 1. Introduction

African swine fever (ASF) is a highly contagious viral disease of domestic and wild swine, with a mortality rate of up to 100% [1, 2]. The causative agent of ASF, the African swine fever virus (ASFv), is a large DNA virus with icosahedral symmetry and the only member of the family Asfarviridae [1]. ASF has been devastating to the global pork industry, causing major socioeconomic burdens due to food security and biodiversity threats [2]. Despite severe impacts, no effective vaccine is currently available to control ASF in endemic areas [3, 4, 5].

ASF was first reported in East Africa in the early 1920s as an acute hemorrhagic fever that caused the death of almost all infected domestic pigs [6, 7]. ASF has remained endemic in Africa, affecting up to 35 African countries [8]. Since the introduction of ASF into Georgia from Africa in 2007, ASF has rapidly spread to eastern Europe, Russia [9], China, other Asian countries, and western Europe [2, 10, 11]. The 2021 outbreak of ASF on the Caribbean island of Hispaniola has caused ASF-free countries, including the United States and Canada, to dedicate their full attention to the prevention of ASF entry into their regions [12].

The United States and Canada are major pork exporters to regions across the globe resulting in large shipments of pigs across borders [13, 14]. An ASF outbreak in the United States or Canada could lead to trade restrictions and other severe consequences to the swine industry and economy in both countries. Therefore, improving surveillance programs using a variety of detection and diagnostic strategies is crucial to prevent further spread of ASF into nonendemic areas. Sampling blood from individual pigs in commercial pig farms is a standard procedure of current ASF surveillance in North America; however, this method is labor-intensive and costly. As a complementary sample matrix for ASF surveillance, aggregate oral fluid (OF) samples collected in pig pens have provided promising results. This includes reliable detection of endemic swine pathogens as suggested in the study by Rotolo et al. [15], in which OF samples were collected in every pen repeatedly over 9 weeks for detecting porcine reproductive and respiratory syndrome virus (PRRSV) in newly introduced replacement gilts from an endemically infected herd. In a study by Olsen et al. [16], the probability of detection of PRRSV using RT-PCR on OF samples was greater than 70% when within-pen prevalence was 20% or more. Promising results were also reported for porcine circovirus type 2 (PCV-2), swine influenza virus (SIV), and *Mycoplasma hyopneumoniae* when sampling occurred every 2 weeks for pigs 5–21 weeks of age [17]; and in a separate study, SIV was detected in OFs when all six pigs in each of the four pens were inoculated [18]. OF collection can be performed by nonveterinary staff with minimal on-site resources and disruption to the animals. In contrast, whole blood or tissue collection requires additional materials and highly trained staff. Therefore, pen-based OF collection for active ASF surveillance may be an efficient, noninvasive alternative that can save resources and time [19].

Oral ingestion of virus particles is the main route of ASFv transmission in domestic pigs [20, 21]. Following oral ingestion, ASFv replicates in the tonsils and oropharynx [22, 23]. Therefore, virus may be detected in pharyngeal swabs and OF at early stages of infection [24]. This suggests OF could be an ideal sample to detect ASFv prior to onset of clinical signs. In previous experimental studies utilizing small groups of pigs inoculated with highly virulent ASFv Georgia 2007/1, ASFv genomic material was detected in OF before the animals developed any noticeable clinical signs; and pigs continued to chew on the collection ropes daily until they developed severe clinical signs [25, 26, 27]. Our previous work evaluated the utility of pen-based aggregate OF testing in pens where larger groups (20–25 pigs/pen) were housed. Only one animal per pen was experimentally infected with either the highly virulent ASFv Georgia 2007/1 or the moderately virulent ASFv Malta 78 to cause gradual transmission to pen mates [28]. Findings provided promising insights on the utility of aggregate OFs as an effective and reliable sample for ASFv detection [28]; however, the diagnostic sensitivity of the aggregate OFs has not yet been fully characterized in a field setting. The current study was performed to further evaluate aggregate OFs for ASF detection using samples collected from Romanian farms with an ongoing ASF outbreak.

## 2. Materials and Methods

### 2.1. Sample Collection on ASF Suspect Farms

The animal sample collection was conducted under the approval of the Institutional Animal Care and Use Committee (PIADC IACUC 262.02-19-P).

Three farms, identified as Farms 1, 2, and 3, reported ASF clinical signs on January 8, 2021; August 6, 2021; and August 27, 2021, respectively. The collection of individual blood from live animals and lymphoid tissues from dead animals was performed on August 4, 2021, 7 months after the initial recognition of ASF signs on Farm 1; on August 9, 2021, 3 days after the initial recognition of clinical signs on Farm 2; and on August 30, 2021, and October 9, 2021, either 13 or 43 days, respectively, after the initial recognition of clinical signs on Farm 3. Aggregate OF samples were collected using cotton ropes (TEGO® Swine Oral Fluid kit, ITL Biomedical, Reston, USA) in each pen of the three farms as previously reported [28]. At each collection, two cotton ropes were hung before feeding on the front gate of the pen approximately 4 feet apart at the shoulder height of the pigs for 30 min without training the pigs to the ropes. After a 30-min period, the ropes were collected, and OF was squeezed out of each rope and pooled into one 50 mL centrifuge tube. These collected OF samples were aliquoted without centrifugation and saved at −70°C until further processing. In some pens, all pigs presented with advanced signs of ASF sickness such as laying down, not moving, and not being able to chew the rope. In these pens, OF collection was not possible.

Individual blood and OF samples were collected from a subset of pens on each of the three farms. Individual blood samples were collected in serum collection tubes (BD Vacutainer SST Tubes, 8.5 mL) and stored at −80°C until used for viral DNA extraction. After allowing 30 min for the pigs to chew on a cotton rope in each pen, the OFs were squeezed out of each rope by hand or using a syringe and collected into one 50-mL centrifuge tube. OF samples from live pigs and tissue samples from pigs that died of ASF-suspected sickness were collected and stored at −80°C until used for viral DNA extraction. Clinical observation of all animals in each pen was carried out by veterinarians and recorded as the number of healthy, sick, and dead pigs in each pen.

### 2.2. Oral Fluid Sample Collection on ASF-Free Farms

A total of 50 aggregate OF samples were collected from farms in Iowa, United States, as described above. The United States has no history of ASF; therefore, these samples were considered true ASF-negative samples.

### 2.3. Nucleic Acid Extraction and Real-Time PCR (RT-PCR)

Total nucleic acid extraction from OFs, whole blood, and tissues was performed using Applied Biosystems™ MagMAX™ CORE nucleic acid purification kit (Thermo Fisheri Scientific Inc.) per kit instruction. VetMAX™ Xeno™ Internal Positive Control RNA (Thermo Fisheri Scientific Inc.) was spiked into the lysis buffer during the extraction process at 2 *μ*L/reaction to serve as an exogenous internal positive control (IPC).

ASFv genomic DNA was detected using a quantitative real-time PCR assay targeting a highly conserved region of the p72 open reading frame [24]. Both primer sequences were modified by a single base pair replacement based on current BLAST analysis (Forward: 5′ C**T**T CGG CGA GCG CTT TAT CAC 3′, Reverse: 5′ GGA AA**T** TCA TTC ACC AAA TCC TT 3′). Taqman® Fast Virus 1-Step Master Mix (Thermo Fisheri Scientific Inc.) was used for the PCR reaction at a volume of 6.25 *μ*L per reaction. VetMAX™ Xeno™ Internal Positive Control–LIZ™ assay was used to detect the IPC at 1 *μ*L/reaction. The PCR reactions were amplified using Applied Biosystems™ QuantStudio™ 5 Real-Time PCR platform. The thermal cycling conditions included an initial denaturation stage at 95°C for 20 s followed by 45 cycles of annealing/extension at 95°C for 10 s and 60°C for 30 s.

### 2.4. Statistical Analysis to Evaluate Two Different Diagnostic Processes with No Gold Standard

To estimate the diagnostic sensitivity of the individual blood collection and testing process for determining pen-level disease status (hereafter referenced as the blood diagnostic process) and the aggregate OF sample collection and testing process (hereafter referenced as the OF diagnostic process), ASF real-time PCR testing results, combined with any attempts to obtain samples that could not be tested, were analyzed using a Bayesian latent class model approach [29, 30]. Because failure of the pigs to provide an OF sample when a rope is hung in a pen contributes to a reduction in the diagnostic capability of this sample type, pens in which a rope was placed but the pigs did not chew on the rope were included as nonpositive results for the OF diagnostic process. This occurred in two pens during this study. If blood samples were collected and were unable to be tested, this would be included as a nonpositive result for the blood diagnostic process; but this did not occur during this study.

Three subsets of data were considered during the analysis, providing three different scopes. These scopes included (1) pens in which a rope was placed and from which at least one pig in the pen had a blood sample collected, (2) pens in which a rope was placed and from which at least 35% of pigs in the pen had blood samples collected, and (3) pens in which a rope was placed in the pen and from which all pigs in the pen had blood samples collected. Results are presented below only for the first scope in which pens with at least one pig sampled by blood and a rope placed for collection of an OF sample.

A Bayesian latent class model was used to estimate sensitivity for two conditionally independent diagnostic processes from three independent populations. Because the pens are the test subjects in this study, the model also provided estimates of the among-pen prevalence for each farm. This model assumes each diagnostic method has a single unknown and uncertain diagnostic sensitivity regardless of disease prevalence among pens.

The model was set up as follows.

(1) $$\begin{array}{c} \begin{array}{cc} Y_{k} & ∼\text{Multinomial}\left(n_{k},\rho_{k}\right)\\ \rho_{k,1} & =\pi_{k}\eta_{1}\eta_{2}+\left(1-\pi_{k}\right)\left(1-\theta_{1}\right)\left(1-\theta_{2}\right)\\ \rho_{k,2} & =\pi_{k}\eta_{1}\left(1-\eta_{2}\right)+\left(1-\pi_{k}\right)\left(1-\theta_{1}\right)\theta_{2}\\ \rho_{k,3} & =\pi_{k}\left(1-\eta_{1}\right)\eta_{2}+\left(1-\pi_{k}\right)\theta_{1}\left(1-\theta_{2}\right)\\ \rho_{k,4} & =\pi_{k}\left(1-\eta_{1}\right)\left(1-\eta_{2}\right)+\left(1-\pi_{k}\right)\theta_{1}\theta_{2} \end{array} \end{array}$$

### 2.5. Parameters

(2) $$\begin{array}{c} \begin{array}{cc} \eta_{l} & ∼\text{Beta}\left(\alpha_{\eta_{l}},\beta_{\eta_{l}}\right)\\ \theta_{l} & ∼\text{Beta}\left(\alpha_{\theta_{l}},\beta_{\theta_{l}}\right)\\ \pi_{k} & ∼\text{Beta}\left(\alpha_{\pi_{k}},\beta_{\pi_{k}}\right) \end{array}, \end{array}$$

where k indexes the independent populations, in this case, the three farms; *ℓ* indexes the conditionally independent diagnostic processes, in this case, there are two, the blood diagnostic process and the OF diagnostic process; *n*_k_ is the number of eligible pens from the farm k; and **Y**_**k**_ is the four-element multinomially distributed vector, with the four elements corresponding to the counts of pens that were:

1. positive on both blood and OF (Blood+/OF+)
2. positive on blood and negative on OF (Blood+/OF−)
3. negative on blood and positive on OF (Blood−/OF+)
4. negative on both blood and OF (Blood−/OF−)

**ρ**_**k**_ is the four-element vector of probabilities of pens falling into each of the previous categories (Blood+/OF+; Blood+/OF−; Blood−/OF+; and Blood-/OF−), *π*_k_ is the pen-level prevalence on farm k, *η*_l_ is the diagnostic sensitivity of process *ℓ*, *θ*_l_ is the diagnostic specificity of process *ℓ*, and *α*_*η*_l__, *β*_*η*_l__, *α*_*θ*_l__, *β*_*θ*_l__, *α*_*π*_k__, *β*_*π*_k__ are all hyperparameters, described in Table 1.

## 3. Results

### 3.1. Overall Results from Three Farms with ASF-Suspected Pigs

Summary statistics by farm and overall for the 203 pens that had at least one pig sampled for testing by the blood diagnostic process and in which a rope was placed in a pen to collect an OF sample for testing are provided in Table 2. The mean (range) numbers of pigs per pen that qualified and were included in the analysis for Farms 1, 2, and 3 were 50.4 (32–52), 14.5 (6–16), and 12.1 (10–13), respectively. The mean (range) percentages of pigs sampled for individual blood testing per pen in Farms 1, 2, and 3 were 58% (40%–100%), 23% (6%–100%), and 35% (8%–100%), respectively. The mean prevalence of pig-level infection within pens included in the analysis was 0.11, 0.02, and 0.39 in Farms 1, 2, and 3, respectively (Table 2). The pen-level prevalence of ASF (proportion of eligible pens infected) in Farms 1, 2, and 3 was 0.44, 0.05, and 0.69, respectively (Table 2).

### 3.2. Evaluation of Two Diagnostic Testing Processes with No Gold Standard

**Figure 1:**
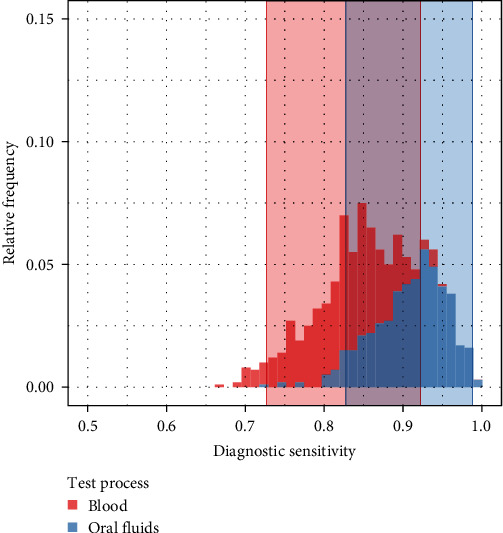
Estimated posterior distributions for the diagnostic sensitivity values for the blood and oral fluid diagnostic processes are displayed as red and blue histograms, respectively. The shaded regions in red and blue depict 95% uncertainty intervals for the diagnostic sensitivity values for each process, respectively.

Using a Bayesian latent class model analysis, the pen-level prevalence of ASF on each farm and the diagnostic sensitivity and specificity of the blood diagnostic process and the OF diagnostic process were estimated at the pen-level (Table 3). Additionally, an estimate of the difference between the diagnostic sensitivity of the pen-level blood diagnostic process and that of the OF diagnostic process was computed to determine if there was a significant distinction between the sensitivities of the two diagnostic processes (Table 3). The estimated diagnostic sensitivity of the OF process did not significantly differ from that of the blood process, as the 95% uncertainty interval estimate of the difference in sensitivity estimates, *η*_2_ − *η*_1_, (−0.04, 0.21), included zero (Figure 1).

To decrease the variability in the diagnostic sensitivity of the pen-level blood diagnostic process and to better meet assumptions of the analysis, a subset of the data was created in which at least 35% of the pigs in each pen were bled and tested and a rope was hung (87 pens across all three farms). Setting a cutoff at 35% allowed the detection of seven or more infected pigs when pigs were randomly sampled, utilizing only 15% of the full dataset. To further strengthen these criteria, another subset of cases was created where 100% of the pigs in each pen were bled and tested and a rope was hung (15 pens across all three farms). Those pens with 100% of pigs bled and tested represent the gold standard but with varying diagnostic sensitivity because of varying within-pen prevalence. The more restrictive criteria greatly reduced the data available for analysis; therefore, the precision of the estimates was sacrificed. Irrespective of the scope of pens considered, the outcome remained consistent: There was no significant difference in the diagnostic sensitivity values of the blood and OF diagnostic processes.

### 3.3. Characteristics of ASF-Affected Pens from Farm 1

A total of 15 of the 34 pens meeting the criteria for this study on Farm 1 had positive pen-level PCR results by at least one of the processes: 13 pens out of 15 tested positive by OF, and 12 pens out of 15 tested positive by blood (Table 4). In one pen, a rope was hung, but the pigs did not chew on the rope. This was included as a negative for the OF diagnostic process; the pen was positive for the blood diagnostic process. Sampling effort for the blood diagnostic process ranged from 41% to 100% in pens on Farm 1 that were positive by either process. The PCR threshold cycle (Ct) values of the 13 positive OF samples were between 30.4 and 37.1, which are considered moderate to weak positive results in the real-time PCR method used in this study. However, Ct values of individual blood samples from those same pens were between 17.3 and 37.7, indicating a wide variation in viremia levels in different pigs even within the same pen. The remaining 19 pens on Farm 1 in which there was an attempt to collect samples for both blood and OF had no positives by either process. Pen sampling effort for the blood diagnostic process ranged from 40% to 48% in pens on Farm 1 in these negative or undetected pens. Clinical observation results at 3 months after the initial recognition of ASF-suspect pigs by experienced veterinarians were recorded as normal for 10 out of the 15 pens that tested positive either by OF, blood, or both.

There were two pens that tested positive by individual blood samples but were negative by the OF diagnostic process. One of the two pens had a very high rate of sick or dead pigs, and the other had no clinical signs observed, with an estimated 16% prevalence based on a sampling rate of about 48% of the pigs in the pen. Among the pens tested, three were positive by the OF diagnostic process but negative by the blood diagnostic process. In two of those three pens, less than 50% of the pigs in the pen were sampled for the blood diagnostic process. In the third pen, all 51 pigs were bled and tested.

### 3.4. Characteristics of ASF-Affected and Neighboring Pens from Farm 2

Five out of the 101 pens included in the study yielded positive results for one or both PCR-based diagnostic processes: three out of five by OF and three out of five by blood (Table 5). Sampling effort for the blood diagnostic process ranged from 31% to 100% in pens on Farm 2 that were positive by either process. The Ct values of three positive OF samples ranged between 32.8 and 36.8, which are moderate to weak positive results. Ct values of individual blood samples from those same pens ranged between 21.0 and 36.9, indicating moderate variation in viremia levels among different pigs even within the same pens. Clinical observation 3 days after the initial recognition of ASF-suspected signs found 0%–100% of pigs as sick or dead, indicating a wide pen-dependent variation in clinical manifestation within the same barn compared to observations on Farm 1, which were made 3 months after the initial recognition of ASF-suspected pigs. Three pens had 25% or more pigs observed with clinical signs but yielded no positive results with the OF or blood diagnostic processes (data not shown because OF sampling was not attempted in these pens). The remaining 96 pens included in the analysis from Farm 2 had no positive detection by either diagnostic process. Sampling effort for the blood diagnostic process in pens on Farm 2, where no positive detections occurred, ranged from 6% to 100%.

Two pens tested positive with the blood diagnostic process but did not test positive by the OF diagnostic process. In one of these two pens, there was a high rate of terminally sick and dead pigs; in the other pen, one out of 15 pigs exhibited clinical signs, and the prevalence was estimated to be approximately 20% based on a 33% sampling effort. Two pens tested positive by the OF diagnostic process, but none of the sampled pigs tested positive by individual blood testing. In one of these two pens, less than a third of the pigs in the pen were sampled; in the other, all of the 12 pigs in the pen were sampled.

### 3.5. Characteristics of ASF-Affected Pens from Farm 3

A total of 47 of the 68 pens included in the study had positive PCR results. Of the 47 pens with positive PCR results, 45 were detected through OF and 41 through individual blood samples (Table 6). Sampling effort for the blood diagnostic process ranged from 17% to 100%, with most pens having 42% (5/12 pigs) of pigs sampled. These pens were positive by either process. The Ct values of the positive OF samples were between 22.3 and 36.7, indicating a range from strong to weak positive results. Ct values of individual blood samples from those same pens were between 15.4 and 36.6, indicating a wide variation in viremia levels in different pigs even within the same pens. The range of estimated ASF prevalence in different pens based on selecting a subset of pigs for blood testing was between 0% and 100%, indicating high variation in pen ASF prevalence in the same farm. Clinical observations of sick or dead pigs at either 13 or 43 days after the initial recognition of ASF-suspected signs were recorded in only five of 68 pens, with a range of 8%–10% (one pig) affected pigs per pen. The remaining 21 pens from Farm 3 had no pigs testing positive for ASF by either diagnostic process. Regarding pen sampling effort, it ranged from 15% to 42%, with most pens having two out of 13 pigs sampled.

There were two pens that tested positive with the blood diagnostic process but were not positive with the OF diagnostic process. The estimated within-pen prevalence values for those two pens were 50% or greater, but the sampling effort was very low: only 17% and 25% (two and three pigs, respectively). There were six pens that tested positive by the OF diagnostic process, but none of the sampled pigs tested positive by individual blood testing. In four of the six pens, only 17% (two pigs) of the pigs in the pen were sampled; and in the other two pens, 42% (five pigs) of the pigs in the pen were sampled. None of the eight pens with nonmatching test results had any pigs with clinical signs.

### 3.6. Diagnostic Findings in Oral Fluid Samples Collected from ASF-Free Areas

Of the 50-pen known negative OF samples from the United States, zero tested positive for ASF with the real-time PCR assay used in this study. This suggests excellent diagnostic specificity for the OF diagnostic process.

## 4. Discussion

The continuous spread of ASFv throughout much of the world has made the control of ASF a global animal health priority, with an increased emphasis on enhancing preparedness to prevent, detect, and respond to a potential outbreak of ASFv [2]. Establishing the strategies to enhance ASF surveillance and diagnostic capacity is crucial for effective response and eradication of the disease in case of entry into the United States or Canada. Compared to individual animal sampling of blood, pen-based aggregate OF collection for active surveillance is a noninvasive alternative requiring less labor and training. High diagnostic performance and reliability of pen-based aggregate OF when pens were sampled repeatedly over time or when the majority of pigs were inoculated with the pathogen were reported for major porcine pathogens such as PRRSV [15, 16], SIV [18], PCV-2, and *M. hyopneumoniae* [17].

To evaluate the diagnostic characteristics of rope-based aggregate OF collection for detection of ASFv, three independent farms reported with ASF-suspected pigs were used to collect individual pig blood samples and an aggregate OF sample from partial pens on each farm. The initial design of this study was to collect both aggregate OF and individual blood samples from all pigs in the pens with varying pen populations and ASF prevalence. However, identifying ASF-positive farms and collecting all individual blood samples with the farm owners' permission were not achievable within the 12-month time frame for this project. Due to those challenges, attempts to collect both OF and blood samples occurred in a total of 203 pens across three farms with only 15 of those 203 pens having a 100% collection rate of individual pig blood samples. Individual blood samples were successfully collected from 35% or more individual pigs in 87 of the 203 pens.

Diagnostic sensitivity of a diagnostic process for detecting disease in a pen of animals is a combination of the probabilities of the infectious agent being contained in the sample and samples containing the infectious agent testing positive. The diagnostic sensitivity for the process of using individual blood tests to determine status of a pen of size *N* can be calculated [31] as a function of the sampling effort, the number of infected pigs in the pen, and the diagnostic sensitivity of the individual blood test on a single infected pig. As an example, when all pigs are tested from a pen of pigs containing one infected pig, the diagnostic sensitivity of this pen-level sampling and testing process is equal to the diagnostic sensitivity of the individual pig test. Even if the PCR test for an individual pig is taken to have a diagnostic sensitivity of 99% at the animal-level, sampling a subset of pigs from the pen results in a less-than-perfect pen-level diagnostic process. If 25 of 50 pigs are selected and tested individually with an individual animal test with perfect diagnostic sensitivity, the pen-level diagnostic process will have a diagnostic sensitivity of 49.5%, assuming there was one infected pig in the pen. The diagnostic sensitivity of the individual blood testing process in this case would not reach over 95% until there were five infected pigs in the pen.

The Bayesian latent class model was chosen to estimate diagnostic sensitivity for the two pen-level diagnostic processes because it does not require a gold standard test [29, 30]. It does, however, assume constant diagnostic sensitivity and specificity for the tests in the analysis. This assumption is likely not true for two reasons. First, diagnostic sensitivity is expected to vary as within-pen prevalence changes; and, second, it is known to be violated for the blood testing process used to make a diagnosis at the pen-level because a varying subset of pigs in each pen were taken rather than a consistent percentage of pigs in each pen. The Bayesian latent class model results are conditional on the actual disease prevalence observed and the sampling effort applied in these farms.

Given the varying diagnostic sensitivity of both processes, the Bayesian latent class model estimates of the sensitivity of these processes are an average over the sampling and within-pen prevalence levels that occurred in this specific study, which makes generalizing to other situations difficult. Within the context of these farms, the diagnostic sensitivity estimate of the OF diagnostic process was not significantly different from the diagnostic sensitivity estimate of the pen diagnosis using the varying subset of individual blood samples from pigs within each pen collected in this study.

There were 11 pens that tested positive by OF diagnostic process but that had no pigs test positive with individual blood PCR testing. Of these, nine pens had less than half of the pigs in the pen sampled for individual blood testing. The diagnostic sensitivity of the blood sampling process was less than 50% (four were between 40% and 50%, one was 31%, and the remaining four were 17%) for all nine of these pens due to the low percentage of pigs sampled and tested (calculations assume there was only one infected pig in each of the pens). The lack of complete sampling (i.e., 100% of pigs with blood samples tested) provides an incomplete picture because the true status of these pens was uncertain, as these pens may have had positive pigs that were not sampled. The other two pens that were positive by the OF diagnostic process but had no sampled pigs test positive by the individual blood diagnostic process did have all pigs in the pen blood tested. The diagnostic sensitivity of the blood testing process would have been about 0.99 for those two pens. These two results could have occurred due to preclinical transmission detected in OF but not in blood, environmental contamination, or random chance.

The blood diagnostic process detected ASFv in six pens in which the OF diagnostic process did not detect the virus. The within-pen prevalence in these pens ranged from 16% to 100%, though the percentages of the pens sampled for the blood diagnostic process ranged widely as well, from 16% to 59%. In two of the pens, the OF diagnostic process did not detect the virus because the pigs did not provide an OF sample, possibly because they were too sick to chew the ropes, although in one pen, there were two apparently healthy pigs.

The limited pen numbers, pen sizes, and estimated within-pen prevalence levels included in this study are insufficient to fully characterize the diagnostic sensitivity of the aggregate OF samples. Determining the fitness for purpose of aggregate OF samples for ASFv detection in a field study where variation in within-pen prevalence cannot be controlled requires blood testing of every pig in every pen with an OF sample to determine the true status (infected or not) of each pen.

Four experimental studies provided promising results regarding the utility of aggregate OF as a potentially effective and reliable sample type for diagnosing ASFv during an outbreak in North America [28]. The findings in the current study, using naturally infected ASF outbreak farms, further suggest that pen-based OF samples may be used to supplement traditional sample types for some surveillance use cases during an ASF outbreak. However, it will be prudent to further evaluate data in ASF endemic areas before making the crucial decision to replace a currently approved individual blood and tissue-based testing method with an aggregate OF method. If enough field validation data are provided through further studies, the OF-based method would be highly valuable for expanding ASF surveillance and response activities because of its ability to test multiple animals with one specimen and the convenience of noninvasive sample collection.

Furthermore, in this study, all three farms had high between-pen variation and within-pen variation of detectable infection, indicating the slow transmission of ASFv after its entry into farms and pens. Different clinical findings between Farm 2 and the two other farms could be related to earlier sampling days after the initial recognition of ASF-suspected signs. This suggests that in an outbreak situation, ASF surveillance based on clinical observation may not be reliable without collection of specimens, and sensitive real-time PCR testing, from both sick and apparently healthy pigs in a pen.

The full genome sequences identified in this study were genotype II Eastern European Georgia 2007/1-like isolates (data not shown). Further validation in different areas involving varying genotypes and pathogenicity of ASFv strains will be important to confirm the results in this study. In conclusion, the analyses of data from three different farms showed no significant difference between the diagnostic sensitivities for real-time PCR using aggregate OF and pen-level determination using real-time PCR of individual blood samples when pens were sampled at varying levels. Although only a subset of pigs per pen were blood tested with varying subsets, the estimated within-pen prevalence values varied, and pen sizes were small, this study presents the first field evaluation on aggregate OF samples for ASFv real-time PCR diagnosis using farms with a current, naturally occurring ASF outbreak. Until satisfactory results from further studies with sufficient sample sizes from a variety of pen sizes with low ASFv prevalence are obtained, the method of using aggregate OF should be recommended as a supplementary sample type for ASF diagnosis in addition to currently National Animal Health Laboratory Network-approved sample types, namely, blood and the spleen, tonsil, and lymph node tissues.

## Figures and Tables

**Table 1:** Parameterization of the Bayesian model.

Parameter	Value	Mode	Source
k	3	—	The three farms

*ℓ*	2	—	Blood and OF diagnostic processes

*Y*_k_	—	—	See Table 2 for counts of pens

*η*_1_	Beta (1, 1)	All values equally likely	Agnostic priors for sensitivity for the diagnostic processes
*η*_2_	Beta (1, 1)		

*θ*_1_	Beta (100, 1)	0.99	Assumed nearly perfect specificity for the diagnostic processes
*θ*_2_	Beta (100, 1)	0.99	

*π*_1_	Beta (16, 20)	0.44	See Table 2 for data informing prior estimates of prevalence by the farm
*π*_2_	Beta (6, 97)	0.06	
*π*_3_	Beta (48, 22)	0.69	

**Table 2:** Descriptive statistics by farm.

Farm	Total pens	Blood^1^+ OF^2^+	Blood+ OF−	Blood−OF+	Blood−OF−	Mean (range) percentage of pigs bled per pen	Mean within-pen prevalence	Mean (range) pen size	Pen-level prevalence
1	34	10	2	3	19	58 (40–100)	0.11	50.4 (32–52)	0.44
2	101	1	2	2	96	23 (6–100)	0.02	14.5 (6–16)	0.05
3	68	39	2	6	21	35 (8–100)	0.39	12.1 (10–13)	0.69
Total	203	50	6	11	136	33 (6–100)	0.16	19.7 (6–52)	0.33

^1^Making a diagnostic determination on the disease status of a pen by sampling a subset of pigs from the pen and PCR testing blood samples taken from the individual pig(s) selected. ^2^Making a diagnostic determination on disease status of a pen by allowing pigs to self-select chewing on a rope hung in their pen and PCR testing of OF samples collected from the rope. Failure of pigs to provide an oral fluid sample was considered a negative or not detected result.

**Table 3:** Output from the Bayesian latent class model.

Description	Parameter	Point estimate	95% uncertainty intervals
Pen-level prevalence			
Farm 1	*π*_1_	0.44	(0.32, 0.56)
Farm 2	*π*_2_	0.04	(0.02, 0.07)
Farm 3	*π*_3_	0.69	(0.61, 0.77)
Diagnostic sensitivity			
Blood^1^	*η*_1_	0.83	(0.73, 0.92)
OF^2^	*η*_2_	0.91	(0.83, 0.99)
Diagnostic specificity			
Blood^1^	*θ*_1_	0.99	(0.97, 1.00)
OF^2^	*θ*_2_	0.99	(0.97, 1.00)
Difference between sensitivities	*η*_2_ − *η*_1_	0.08	(−0.04, 0.21)

^1^Determining the disease status of a pen diagnostically involves sampling a subset of pigs from the pen and PCR testing blood samples taken from these selected individuals. ^2^Determining the disease status of a pen diagnostically involves allowing pigs to self-select by chewing on a rope hung in their pen and PCR testing oral fluid samples collected from the rope. Failure of pigs to provide an oral fluid sample was considered a negative result.

**Table 4:** Diagnostic findings of ASF-affected pens in Farm 1.

Pen ID	OF^1^ PCR Ct	Blood PCR Ct range	Percentage of sampling effort (#sampled/#pigs in pen)	Percentage of positive blood (#positive/#sampled)	Percentage of sick or dead pigs (#sick or dead/#pigs in pen)
H1-P2	36.9	34.6	41.2 (21/51)	4.8 (1/21)	0.0 (0/51)
H2-P25	36.3	Neg	48.1 (25/52)	0.0 (0/25)	0.0 (0/52)
H2-P26	32.1	20.2–35.6	100.0 (47/47)	29.8 (14/47)	8.5 (4/47)
H2-P27	Neg^2^	17.1–29.3	59.4 (19/32)	100.0 (19/19)	93.8 (30/32)
H2-P28	33.5	17.3–37.2	100.0 (50/50)	58.0 (29/50)	6.0 (3/50)
H2-P29	36.3	19.2–35.3	94.2 (49/52)	4.1 (2/49)	0.0 (0/52)
H2-P30	37.1	Neg	100.0 (51/51)	0.0 (0/51)	0.0 (0/51)
H2-P31	35.8	21.4–36.7	100.0 (51/51)	5.9 (3/51)	2.0 (2/51)
H2-P32	35.5	20.1–36.7	100.0 (51/51)	17.6 (9/51)	3.9 (2/51)
H2-P36	36.6	22.5–30.2	46.2 (24/52)	8.3 (2/24)	0.0 (0/52)
H2-P37	37.0	19.2	46.2 (24/52)	4.2 (1/24)	0.0 (0/52)
H2-P39	30.4	17.9–36.8	100.0 (44/44)	84.1 (37/44)	0.0 (0/44)
H2-P40	33.3	20.9–37.7	100.0 (48/48)	25.0 (12/48)	0.0 (0/48)
H2-P41	Neg	18.6–34.9	48.1 (25/52)	16.0 (4/25)	0.0 (0/52)
H2-P42	36.4	Neg	46.2 (24/52)	0.0 (0/24)	0.0 (0/52)

^1^OF denotes aggregate oral fluid (OF) collected using the rope in each pen. ^2^No collection and testing of OF in H2-P27 were assumed due to most pigs showing advanced stage of ASF signs including a mortality rate of 40.6%. For statistical analysis, this pen was included as OF diagnostic process negative.

**Table 5:** Diagnostic findings of ASF-affected and neighboring pens in Farm 2.

Pen ID	OF^1^ PCR Ct	Blood PCR Ct range	Percentage of sampling effort (#sampled/#pigs in pen)	Percentage of positive blood (#positive/#sampled)	Percentage of sick or dead pigs (#sick or dead/#pigs in pen)
H1-C11-B26	36.8	Neg	31.2 (5/16)	0.0 (0/5)	12.5 (2/16)
H1-C11-B33	Neg	21.2	33.3 (5/15)	20.0 (1/5)	6.7 (1/15)
H1-C11-B36	32.8	21.2–36.9	100.0 (16/16)	50.0 (8/16)	56.2 (9/16)
H1-C11-B37	Neg^2^	21.0–26.2	50.0 (7/14)	100.0 (7/7)	100.0 (14/14)
H1-C11-B38	33.8	Neg	100.0 (12/12)	0.0 (0/12)	0.0 (0/12)

^1^OF denotes aggregate oral fluid (OF) collected using a rope in each pen. ^2^No collection and testing of OF in H1-C11-B37 were assumed due to all pigs showing advanced stage ASF signs, including a mortality rate of 50%. Seven of the pigs had their spleens sampled and tested. All seven spleen samples tested positive, with PCR Ct values ranging from 15.4 to 16.8.

**Table 6:** Diagnostic findings of ASF-affected pens in Farm 3.

Pen ID	OF^1^ PCR Ct	Blood PCR Ct range	Percentage of sampling effort (#sampled/#pigs in pen)	Percentage of positive blood (#positive/#sampled)	Percentage of sick or dead pigs (#sick or dead/#pigs in pen)
H4-C1-B1	Neg	32.4	16.7 (2/12)	50.0 (1/2)	0.0 (0/12)
H4-C1-B16	32.3	Neg	16.7 (2/12)	0.0 (0/2)	0.0 (0/12)
H4-C2-B2	34.1	Neg	16.7 (2/12)	0.0 (0/2)	0.0 (0/12)
H4-C2-B11	36.7	Neg	16.7 (2/12)	0.0 (0/2)	0.0 (0/12)
H4-C2-B15	33.6	Neg	16.7 (2/12)	0.0 (0/2)	0.0 (0/12)
H4-C3-B9	Neg	16.5–36.3	25.0 (3/12)	66.7 (2/3)	0.0 (0/12)
H5-C1-B7	23.4	19.1–20.8	38.5 (5/13)	100.0 (5/5)	7.7 (1/13)
H5-C1-B10	23.4	19.0–21.0	45.5 (5/11)	100.0 (5/5)	0.0 (0/11)
H5-C1-B16	24.3	18.3–21.3	41.7 (5/12)	100.0 (5/5)	0.0 (0/12)
H5-C2-B2	26.7	19.0–20.4	41.7 (5/12)	100.0 (5/5)	0.0 (0/12)
H5-C2-B4	25.2	19.0–31.7	41.7 (5/12)	100.0 (5/5)	0.0 (0/12)
H5-C2-B8	23.8	17.9–19.9	41.7 (5/12)	100.0 (5/5)	0.0 (0/12)
H5-C2-B10	23.8	17.2–20.2	41.7 (5/12)	100.0 (5/5)	0.0 (0/12)
H5-C2-B14	23.5	15.4–21.8	41.7 (5/12)	100.0 (5/5)	8.3 (1/12)
H5-C2-B15	24.4	15.5–19.6	50.0 (5/10)	100.0 (5/5)	10.0 (1/10)
H6-C1-B2	24.2	20.3–22.1	41.7 (5/12)	100.0 (5/5)	8.3 (1/12)
H6-C1-B3	28.9	21.4–32.1	45.5 (5/11)	100.0 (5/5)	0.0 (0/11)
H6-C1-B4	27.7	21.6	8.3 (1/12)	100.0 (1/1)	0.0 (0/12)
H6-C1-B5	27.3	20.7–27.7	50.0 (6/12)	100.0 (6/6)	0.0 (0/12)
H6-C1-B6	26.5	19.1–25.0	50.0 (6/12)	100.0 (6/6)	0.0 (0/12)
H6-C1-B7	27.4	19.2–27.3	50.0 (6/12)	83.3 (5/6)	0.0 (0/12)
H6-C1-B8	31.3	20.4–21.5	50.0 (6/12)	50.0 (3/6)	0.0 (0/12)
H6-C1-B9	26.2	20.5–25.0	41.7 (5/12)	80.0 (4/5)	0.0 (0/12)
H6-C1-B10	27.9	20.7–29.2	41.7 (5/12)	60.0 (3/5)	0.0 (0/12)
H6-C1-B12	27.1	20.7–22.1	41.7 (5/12)	60.0 (3/5)	0.0 (0/12)
H6-C1-B13	30.7	19.8–24.6	41.7 (5/12)	100.0 (5/5)	0.0 (0/12)
H6-C2-B1	28.5	17.9–23.3	41.7 (5/12)	80.0 (4/5)	0.0 (0/12)
H6-C2-B4	30.0	18.7–36.9	41.7 (5/12)	60.0 (3/5)	0.0 (0/12)
H6-C2-B5	27.8	17.6–17.8	41.7 (5/12)	40.0 (2/5)	0.0 (0/12)
H6-C2-B7	33.7	17.1–35.4	45.5 (5/11)	40.0 (2/5)	0.0 (0/11)
H6-C2-B8	25.2	25.2–36.6	41.7 (5/12)	60.0 (3/5)	0.0 (0/12)
H6-C2-B9	28.2	20.9	41.7 (5/12)	20.0 (1/5)	0.0 (0/12)
H6-C2-B10	28.7	16.9–35.7	41.7 (5/12)	60.0 (3/5)	0.0 (0/12)
H6-C2-B11	32.9	18.1	45.5 (5/11)	20.0 (1/5)	0.0 (0/11)
H6-C2-B13	29.9	20.4	41.7 (5/12)	20.0 (1/5)	0.0 (0/12)
H6-C2-B16	22.3	19.1–20.2	41.7 (5/12)	60.0 (3/5)	0.0 (0/12)
H6-C3-B1	26.2	17.3–33.8	41.7 (5/12)	40.0 (2/5)	0.0 (0/12)
H6-C3-B2	35.1	Neg	41.7 (5/12)	0.0 (0/5)	0.0 (0/12)
H6-C3-B3	30.7	17.1–18.0	45.5 (5/11)	40.0 (2/5)	0.0 (0/11)
H6-C3-B4	28.5	24.2	41.7 (5/12)	20.0 (1/5)	0.0 (0/12)
H6-C3-B6	28.8	21.1–35.0	100.0 (11/11)	18.2 (2/11)	0.0 (0/10)
H6-C3-B7	31.7	19.6–32.9	100.0 (10/10)	20.0 (2/10)	0.0 (0/10)
H6-C3-B9	28.3	20.3	41.7 (5/12)	20.0 (1/5)	0.0 (0/12)
H6-C3-B12	30.3	18.4–25.5	100.0 (11/11)	18.2 (2/11)	9.1 (1/11)
H6-C3-B13	29.8	Neg	41.7 (5/12)	0.0 (0/5)	0.0 (0/12)
H6-C3-B15	28.9	20.2–20.6	41.7 (5/12)	40.0 (2/5)	0.0 (0/12)
H6-C3-B16	27.1	26.2	41.7 (5/12)	20.0 (1/5)	0.0 (0/12)

^1^OF denotes aggregate oral fluid (OF) collected using a rope in each pen.

## Data Availability

Raw sequencing data and reference-guided assembled genomes from this study are available via the National Center for Biotechnology Information at BioProject accession PRJNA991613.
